# Chimeric Antigen Receptor T-Cell Therapy for the Treatment of Melanoma: A Systematic Review of Phase One Clinical Trials

**DOI:** 10.1177/15330338261452884

**Published:** 2026-06-17

**Authors:** Pavan Shet, Prerana Ghosh, Karan Varshney, Sameen Sawdagar

**Affiliations:** 1 60078 Department of Medicine, St.Vincent’s Hospital Melbourne, Fitzroy, VIC, Australia; 2 1456 Department of Medicine, Ballarat Base Hospital, Ballarat Central, VIC, Australia; 3 Department of Medicine, School of Public Health and Preventative Medicine, 2541 Monash University, Melbourne, VIC, Australia; 4 159388 School of Public Health and Preventative Medicine, Second Affiliated Hospital of Nanjing Medical University, Nanjing, Jiangsu, China

**Keywords:** CAR T-cell therapy, adoptive cellular immunotherapy, melanoma, skin cancer, clinical trials, phase one

## Abstract

**Introduction:**

*Melanoma is a growing health concern,* with global incidence increasing annually. However, treatment for refractory disease is currently limited. Chimeric antigen receptor (CAR) T-cell therapy has shown promising results in haematological malignancies. Despite this, its utility in solid cancers, such as melanoma, is not yet established. We performed a systematic review of phase one trials to help determine and describe foundational understandings of the safety and efficacy of CAR T-cell therapy for melanoma.

**Methods:**

This systematic review was conducted in accordance with the Cochrane Collaboration Handbook for Systematic Review of Interventions and the Preferred Reporting Items for Systematic Reviews and Meta-Analysis (PRISMA) Statement Guidelines. Databases were searched to identify articles before November 18, 2025, which described the use of CAR T-cell therapy in melanoma patients with published results. Study, patient, and treatment characteristics were summarised and analysed.

**Results:**

In total, 2726 articles were screened. Ultimately 5 studies comprising 15 patients with melanoma treated with CAR T-cell therapy were analysed and discussed. T-cell therapies were all autologous and the targets for the CAR T-cell therapies included GD2, PD-1 and cMET antigens across a wide variety of cell doses. Across pooled data, clinical responses varied, with some patients displaying either a partial or complete clinical response. Treatment related effects were of grade one or two severity, with no serious adverse effects reported, including neurotoxicity side effects, or treatment limited related toxicity/mortality events. Peak expansion typically occurred within day 7 to 28, but persistence was limited across studies.

**Conclusion:**

CAR T-cell therapy for melanoma is a novel treatment approach in its infancy. Results are preliminary, and remain largely descriptive, as findings are limited by heterogeneity and small sample sizes. Further investigation through additional phase one trials, and subsequent phase two/three trials, are required for better establishing direct clinical viability.

## Introduction

Melanoma, which is caused by the uncontrolled proliferation of melanocytes, is an aggressive skin cancer with rising incidence across the last fifty years.^1^ According to the International Agency for Research on Cancer’s most recent global cancer estimates, approximately 330,000 new cases and 58,000 deaths occur annually, with age standardised incidence and mortality rates greatest in Oceanic populations (29.80 and 2.30 per 100,000 people respectively).^1,2^ This rise is largely attributed to increasing ultraviolet radiation exposure, a potent mutagen of melanocytes, as well as an increasingly aging population and improved diagnostic tools.^1,2^

Current standard practice for the treatment of melanoma includes various forms of surgery, chemotherapy and immunotherapy.^3^ Many patients undergoing these therapies may continue to have disease progression, or develop intolerable side effects, whilst others may not be viable for such treatments.^4,5^ To help address these burdens, there is a significant need for the development of new treatments to improve outcomes in refractory melanoma patients.

Amongst newly developing therapeutic options, adoptive cell-based therapies, such as chimeric antigen receptor (CAR) T-cell therapy, are emerging for the treatment of advanced and treatment resistant solid tumours, including melanoma.^6,7^ Traditional T-cell activation requires antigens to be presented to a major histocompatibility complex (MHC).^8^ Tumour cells can bypass this system by downregulating MHC expression.^9^ CAR-T cells are antigen receptors that are synthetically engineered with features of both T-cell receptors and antibodies, which allows them to bind with targeted tumour specific antigens, independent of MHC expression.^8,10^ This enhanced T-cell specificity can result in potentially powerful anti-tumour effects.

A recent meta-analysis found treatment with CAR T-cell therapy in solid cancers resulted fewer relapse rates and side effects in comparison to haematological cancers; however, overall response rates were only 9-20% in solid cancers versus 71% in haematological cancers.^11,12^ Therefore, studies demonstrating the safety and efficacy of CAR T cell therapy in solid tumours, such as melanoma, are limited.

Preclinical testing of CAR T-cell therapies for melanoma have shown promising results, with many possible cell antigens, such as CD126 and HER2, identified as viable treatment targets.^13,14^ As a result, clinical trials are now slowly being conducted, although study variables vastly differ. There is hence a need to better synthesise the evidence of the various CAR T-cell treatments of melanoma. To the best of our understanding, no such review has been conducted yet. To bridge this gap, we conducted a systematic review of the safety and efficacy of CAR T-cell therapy for the treatment of melanoma to help establish and guide future research.

## Methods

This systematic review was conducted in accordance with the Cochrane Collaboration Handbook for Systematic Review of Interventions and the Preferred Reporting Items for Systematic Reviews and Meta-Analysis (PRISMA) Statement Guidelines.^15,16^

CAR T-cell therapy for melanoma within human trials is a rapidly emerging field, with treatment being quite novel with only phase one clinical trials currently available. In this review, a broad definition of CAR T-cell therapy was adopted to capture the full spectrum of such approaches. This includes classical CAR constructs as well as engineered T-cell approaches in which tumour antigen recognition was mediated, either wholly or partially, by a CAR. In this systematic review, studies were only eligible for inclusion if they were completed with published results. As a review for a novel therapy, to capture the full availability of results, non-peer-reviewed sources, such as pre-prints were allowed. Furthermore, studies needed to be reported in English and display stratified treatment-related outcomes. No restrictions across randomisation, CAR T-cell therapy type, location or date between studies were applied. Exclusion criteria for this review included non-original texts, such as reviews, and commentaries, and laboratory studies including *in vitro* and animal studies with no patient studies. Purely adoptive T-cell receptor therapies without CAR involvement were also excluded.

On November 18, 2025, PubMed, Embase, ClinicalTrials.gov, Cochrane, International Clinical Trials Registry Platform and Medrxiv were systematically searched. Backwards snowball searching, by manually searching the reference list of included articles, was also conducted. This review aimed to understand CAR T-cell therapy as a treatment for melanoma. Therefore, to capture as many eligible articles as possible, the search strategy was broad, including “CAR T-cells,” “melanoma,” and terms closely associated with them such as “autologous T cell” and “malignant skin cancer”. Details of the full literature search are provided in Supplementary Table 1.

After conducting the searches, duplicates were removed, and articles were independently screened by three reviewers according to the inclusion and exclusion criteria. Studies were first screened by their title and abstract and articles that remained were then analysed independently by their full text by three reviewers. Discrepancies were discussed and finalised amongst these three reviewers until consensus were reached regarding the articles to be included in the review. The complete workflow is shown in the PRISMA checklist in Supplementary Table 2. The prospective systematic review protocol was registered on PROSPERO on May 13, 2025 (CRD420251051530). This protocol was edited as it was originally registered as a meta-analysis, however, after screening, and a corresponding lack of patients, data, and insufficient statistical homogeneity for quantitative analysis, the protocol and review were altered to be a systematic review without meta-analysis. Two authors were also added after protocol registration. One was added for guidance due to their methodological expertise in reviews to support the transition from a meta-analysis to a systematic review and the other added author contributed to the screening process to enhance robustness and increase chances of capturing all available viable texts.

Due to heterogeneity of data, low patient population across included studies, and inconsistencies in data reporting of studies, a meta-analysis was not possible for this review. Study, patient, treatment and outcome data were retrieved from each respective study and displayed in tabular format. Study data included authors and their year of publication; patient characteristics consisted of cancer characteristics of the melanoma population; treatment data encompassed information related to CAR T-cell types, production and delivery. Outcome data included information related to treatment response, adverse effects, expansion and persistence.

Data was examined across included studies to investigate safety and efficacy of CAR T-cell therapy in melanoma. Across all treatment, patient, and outcome data, categorical data was grouped according to frequency and ordinal data was collated and analysed hierarchically. This allowed for an effective presentation of the respective information. Continuous variables, where appropriate, were quantitatively analysed using measures of central tendency and dispersion and pooled accordingly for more detailed analysis.

As all included studies were non-randomised studies, the risk of bias quality assessment was conducted using the updated draft Risk Of Bias In Non-randomized Studies of Interventions (ROBINS-I) framework.^17^ This builds on the original ROBINS-I tool by Sterne et al (2016).^18^ This framework was utilised as it incorporates refinements to signalling questions and provides algorithmic guidance for risk of bias assessments. This method was conducted to highlight any bias that may have risen from the body of evidence. The process completed by two reviewers with disagreements resolved through consensus after discussing discrepancies.

## Results

**Figure 1.**
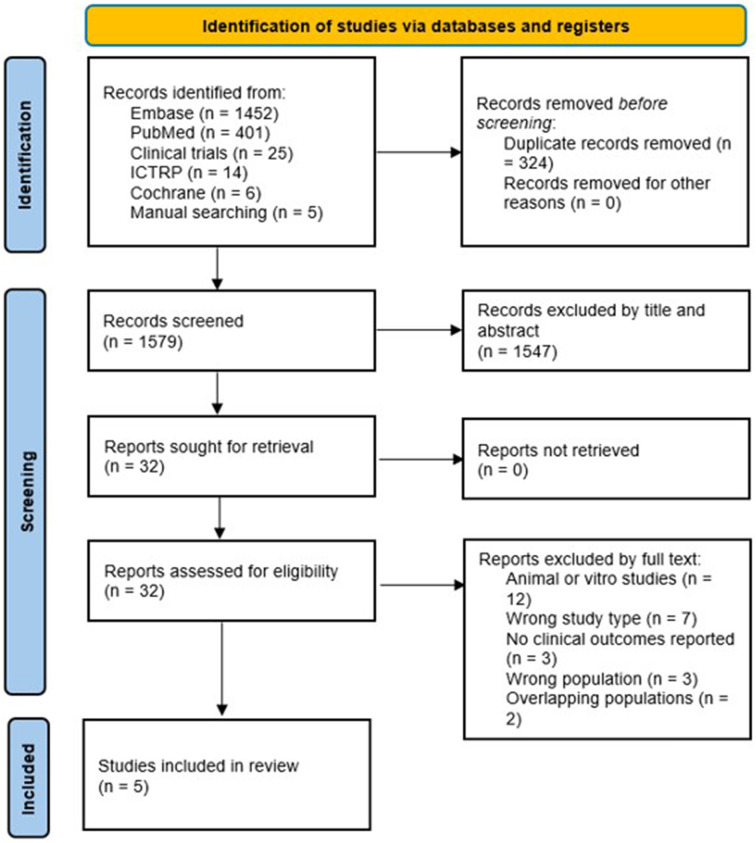
PRISMA 2020 flow diagram

The complete screening process, represented as a PRISMA flowchart, is depicted in Figure 1. The initial search yielded 2726 results. After removal of duplicates between databases and ineligible studies filtered by title and abstract, 86 remained and were fully evaluated for eligibility according to the inclusion and exclusion criteria.

Of the 86 articles that were searched by full text, 5 articles were ultimately included in this review. Of the included results, they all consisted of open label, phase one clinical trials.^19-23^ As per the inclusion criteria, studies needed to have published results with treatment stratified data. Many potentially viable studies were terminated early, completed with unpublished results or had pooled results with other modalities or cancers and therefore excluded. All studies that were included were either peer-reviewed full-texts or conference abstracts. No pre-prints satisfied the inclusion criteria. Only a small number of participants (range 1-7) were recruited in each study. This resulted in a total population of 15 patients being included in this systematic review.

Risk of bias assessment, expressed through the updated draft ROBINS-I framework, is depicted in Table 1. Regarding categorical study quality scores, 60% (n=3) were classified as having low risk of bias,^19,20,22^ 20% (n=1) had moderate risk of bias^23^ and 20% (n=1) had serious risk of bias, which was the study published as a meeting abstract.^21^ This result was largely due to its lack of published information. The most common methodological flaws from the quality assessments included: limited identification of confounding factors and strategies in managing them and study participation bias into intervention groups.

**Table 1.** Updated Draft Risk of Bias in Non-Randomized Studies – Of Interventions Framework

Author (year)	Bias due to confounding	Bias in classification of interventions	Bias in selection of participants into the study	Bias due to deviations from intended interventions	Bias due to missing data	Bias in measurement of the outcome	Bias in selection of the reported result	Overall
Chen et al (2024)	Moderate	Low	Low	Low	Low	Low	Low	Low
Fulbright et al (2020)	Serious	Serious	Moderate	Moderate	Serious	Low	Serious	Serious
Gargett et al (2024)	Moderate	Low	Moderate	Low	Low	Low	Low	Moderate
Shah et al (2023)	Moderate	Low	Low	Low	Low	Low	Low	Low
Zhang et al (2024)	Moderate	Low	Low	Low	Low	Low	Low	Low

Study and patient characteristics of studies are illustrated in Table 2. All studies were published as journal articles with complete results except one, which was represented through a meeting abstract.^21^ The pooled findings across studies are descriptive, as opposed to strictly being estimates of treatment efficacy.

**Table 2.** Patient and Study Characteristics of Included Studies

Study (year)	Melanoma grading	Number of patients	Sex	Age (Mean)	ECOG status (range)	Previous therapy	Concurrent/Pre-treatment	CAR T-cell target	T-cell origin	CAR delivery method
Chen et al (2024)	Grade 4 melanoma	1	Male: 0	55	0-1	Immunotherapy, surgery, chemotherapy, targeted therapy	None	PD-1	Autologous	Lentiviral vector
			Female: 1							
Fulbright et al (2020)	Grade 4 melanoma	3	Unknown	NA	0-2	Standard therapy	None	GD2	Autologous	Unknown
Gargett et al (2024)	Grade 3C/4 melanoma	7	Unknown	Range 35 - 74	0-1	Standard therapy	Targeted therapy, lymphodeple-tion if viable	GD2	Autologous	Retroviral vector
Shah et al (2023)	Grade 4 melanoma	3	Male: 1	49	0-1	Chemotherapy, immunotherapy	None	cMET	Autologous	RNA vector
			Female: 2							
Zhang et al (2024)	Grade 3C melanoma	1	Male: 0	61	0-1	Surgery, chemotherapy, anti-angiogenic therapy	None	PD-1	Autologous	Lentiviral vector
			Female: 1							

ECOG: Eastern Cooperative Oncology Group; PD-1: programmed cell death protein 1; GD2: disialoganglioside 2; cMET: cellular mesenchymal-epithelial transition; CAR: chimeric antigen receptor.

Median study sample size was three with range from one^19,20^ participant to seven.^23^ Patients ranged from 35 to 74 years of age, with an ECOG status between zero to two or zero to one with the exclusion of the meeting abstract^21^; with four studies specifying minimum life expectancy of greater than three months.

Each study recruited patients with melanoma at grade three or higher. Patients also had refractory disease, failing prior treatments, such as surgery and chemotherapy; and at the time of CAR T-cell therapy, were free of any active effects of other treatments. However, one study^23^ permitted ongoing targeted therapy drugs, dabrafenib and trametinib, or pre-treatment with lymphodepletion, for applicable patients.

The targets for the CAR T-cell therapies included programmed cell death protein 1 (PD-1) in two studies (n=2),^19,20^ cellular mesenchymal-epithelial transition (cMET) in one study (n=1)^22^ and disialoganglioside 2 (GD2) in two studies (n=10).^21,23^ Amongst the two GD2 studies, one was reported as a meeting abstract. All studies used autologous T-cell production and CAR T-cell culture time ranged between 9 to 12 days. Cumulative administered T-cell doses ranged between 2.49 x 10^6^ to 1.00471 x 10^9^.

Clinical outcomes and treatment characteristics are summarised in Table 3. In assessing melanoma response to CAR T-cell therapy, four studies used the Response Evaluation Criteria in Solid Tumors 1.1 (RECIST1.1)^19,20,22,23^ whilst one^21^ did not mention the assessment criteria used. Pooled data assessing for clinical response highlighted that four patients (27%) displayed progressive disease (PD), three patients (20%) showed stable disease (SD), and seven (47%) were found to have partial response (PR). Only one patient (7%) in the review reported a complete response (CR), after having had six doses of CAR T-cells targeting PD-1, at 0.57 – 511.8 x 10^6^, each given three months apart; with MRI scans showing a reduction of metastatic paravascular iliac lymph node with target lesions reducing by 77% on MRI. Pooled clinical responses highlight that most patients displayed a positive clinical response; notably the sample size was small and moderately heterogeneous. This was true with or without inclusion of the meeting abstract.^1^

**Table 3.** Treatment Characteristics of Included Studies

Study (year)	No. of patients	Best response (no. Patients)	CAR T-cell dose (/m^2^)	Assessment criteria	Grade 1 or 2 adverse effects	Grade 3 or 4 adverse effects	Peak CAR T-cell expansion	CAR T-cell persistence
Chen et al (2024)	1	PR (1)	1 dose at 2.49 x 10^6^	RECIST1.1	1	0	Day 14	Day 90 <20% of peak
Fulbright et al (2020)	3	PD (2)	1 dose at 2 -4 x 10^8^	Unknown	Unknown	0	Day 7 (18,250 copies/μg)	Month 12 (159 copies/μg)
		PR (1)						
Gargett et al (2024)	7	PD (1)	1 dose at 1 -10 x 10^7^	RECIST1.1	6	0	Day 7-28 (2000 copies/μg)	Week 6
		SD (1)						
		PR (5)						
Shah et al (2023)	3	PD (1)	6 doses at 1 x 10^8^	RECIST1.1	Unknown	0	Failed expansion	Limited
		SD (2)						
Zhang et al (2024)	1	CR (1)	6 doses at 0.57 – 511.8 x 10^6^	RECIST1.1	1	0	Day 7	Day 40

RECIST1.1: Response Evaluation Criteria in Solid Tumors 1.1; PR: partial response; PD: progressive disease; SD: stable disease; CR: complete response; CAR: chimeric antigen receptor.

Pooled characteristics of adverse effects illustrated that four studies (n = 12) reported no dose limiting toxicity and/or mortality.^19,20,22,23^ Of available data, in accordance with the Common Terminology Criteria for Adverse Events (CTCAE) version 5.0^24^ eight (89%) patients reported a grade one or two side effect ranging from mild to minimal respiratory, gastrointestinal, dermatological, haematological and constitutional symptoms. One (11%) patient reported no adverse effects and in six patients this was unknown. All 15 patients (100%) reported no grade three or higher adverse side effects. Cytokine Release Syndrome (CRS) was present in only one (11%) patient,(this was at grade one) with unexplained low-grade fever and arthralgia that resolved after 24 hours. No neurotoxic syndromes, such as Immune Effector Cell-Associated Neurotoxicity Syndrome (ICANS) were reported across the CAR T-cell therapies in the included studies.

Peak CAR T-cell expansion occurred between day 7 to 28 in four studies (n= 12),^19-21,23^ with one study (n = 3)^22^ failing expansion. Two studies (n=10)^21,23^ reported CAR T-cell copies on expansion, of 2000 and 18,250 copies/μg. Of note, the 18,250 figure is from a meeting abstract.^21^ The study showing no expansion also displayed no CAR T-cell persistence.^22^ Of the other remaining studies, persistence varied greatly between them, ranging from 40 days to 12 months post infusion. With the exclusion of the meeting abstract, persistence ranged between 40 to 90 days.^21^

## Discussion

To the best of our knowledge, this is the first review to analyse clinical outcomes of CAR T-cell therapy in patients with melanoma. While this review consists of a small sample size of only 15 patients, this limited sample size was relatively predictable due to the novelty of this therapy. Other likely reasons for the limited sample size were only phase one testing currently being conducted, unestablished and potentially dangerous side-effect profile limiting eligibility in studies, and the high financial costs of conducting CAR T-cell therapy research.^7^ Therefore, despite our results highlighting that most patients developed a partial or complete clinical response, the results are largely exploratory in nature and are limited in the immediate clinical application. This is partially because of the sample in this population being small in size, and the participants come from quite heterogenous datasets. With known clinical response rates, primary end points,(such as objective response rates and disease control rates) can theoretically be established from the findings of these studies. Nevertheless, as the findings consist of multiple single-patient cohorts and heterogeneous trials, they offer little immediate utility. Instead, these results are better analysed descriptively to help establish a foundational discourse for future research to develop more accurate measures of efficacy.

Previous research has shown that poor persistence and expansion of CAR T-cell therapies may be associated with cancer progression, relapse, and/or treatment failure, and therefore, these are important variables to optimise.^25^ In this regard, while data with CAR T-cell therapies for haematological cancer is well understood, understandings of solid cancers such as melanoma are still developing and further research needs to be conducted to ascertain acceptable values of persistence and expansion for adequate therapeutic success. In developing this discourse, our review found Fulbright et al (2020)^21^ developed greatest expansion at 18,250 copies/μg at day 7, however, as results are published in a meeting abstract, conclusions from this study are especially uncertain. Gargett et al (2024)^23^ found that expansion occurred in a range of 7-28 days with approximately 2000 cell copies/μg. Furthermore, there was a similar range found in other studies in this review. Gargett et al (2024)^23^ also found that inflammatory cytokines, such as GM-CSF, CCL2 and IL-8, increased naïve-like T cells in the infusion, and prior lymphodepletion may also increase expansion. Lymphodepletion is currently being tested in an ongoing clinical trial (NCT04897321), and similarly, future studies utilising lymphodepletion, and other comparable methods, could be developed to continue to improve expansion.

Due to inconsistencies in CAR T-cell doses, with no clear effect on therapeutic outcomes, it is uncertain whether CAR T-cell dose influences expansion. However, research suggests that dose optimisation of CAR T-cell treatment - with nuances across dose numbers, fractionalisation and scheduling - may lead to minimalised adverse effects and improved clinical efficacy.^26,27^ Therefore, future studies may benefit from testing this hypothesis and researching if there is an optimal cell dosing strategy for CAR T-cell therapy of melanoma. Continued advancement of CAR engineering characteristics through development of co-stimulatory domains and adoption of later generations may also help increase expansion and warrants further exploration in upcoming trials.

The highest persistence in this review came from Fulbright et al (2020)^21^ with 159 cell copies/μg reported at the 12-month period. Of note, as results were published in a meeting abstract, carrying a serious risk of bias, they should be interpreted with caution. Following Fulbright et al (2020), the next highest persistence was found in Chen et al (2014),^19^ reaching <20% of its peak at 90 days. In comparison to both studies, a recent meta-analysis of CAR T-cell therapy in haematological malignancies found persistence to last many years.^25^ With such large discrepancies, it is a limitation that needs to be addressed before its viable clinical application. Several ongoing animal studies are being developed to potentially increase persistence, including co-expression of CAR T-cells with receptors such IL-15,^28^ knockout of inhibitory signals such as REGNASE-1,^29^ and TOX.^30^ Other engineered CAR techniques, including armoured CAR T-cells, such as Super2 and IL-33-armoured CAR T-cells are also being explored as potential persistence enhancing technique.^31^ Future studies may benefit from implementation of such strategies and others to help address limitations of poor persistence amongst melanoma and other solid cancers.

In terms of adverse effects, our review found CAR T-cell therapy of melanoma to be relatively safe with no severe toxicity observed across studies. In Gargett et al (2024),^23^ one out of the seven patients seven experienced no adverse effects; and of the rest, all reactions were grade one or two, which according to CTCAE, are mild to minimal. There were no grade three or higher (severe or greater) adverse effects, treatment-related toxicity/mortality or any neurological syndromes such as ICANS. Other studies similarly displayed no such adverse side effects, except for Shah et al (2023)^22^ in which one instance of grade one CRS was recorded, which resolved after 24 hours.

While such limited adverse effects may be reassuring, they may be reflected from the low dosages used within the included studies, small patient population, poor expansion from clinical trials, or lack of long-term follow-up. Therefore, safety conclusions of CAR T-cell therapy for the treatment of melanoma need to be interpreted with caution. Furthermore, our review’s results greatly differ from what has been previously described in the literature. A past meta-analysis exploring treatment-related adverse effects across solid neoplasms^12^ revealed that pooled all grade rates of CRS was 35%, and across neurological syndromes, it was 6%. With our review demonstrating no neurological syndromes and only one mild episode of CRS, this disparity warrants further analysis. Future studies could therefore be developed to further categorise and examine treatment-related side effects, leading to a greater understanding of if CAR T-cell technologies for the treatment of melanoma are potentially safer than other cancers.

Additional scope to overcome barriers of the tumour microenvironment, increase clinical response rates, persistence and expansion may also include: continued novel modifications to CAR structure and manufacturing processes, including reducing treatment costs, targeting multiple target antigens, and combination therapies such as the addition of oncolytic viruses, checkpoint immune inhibitors or implementing other immunomodulatory effects to improve the tumour eradicating ability of CAR T-cells.

While it may be too early at this time to establish efficacy benchmarks for CAR T-cell therapy in melanoma, outcomes from related adoptive cellular immunotherapies can provide useful reference points for future studies and reviews. In early 2024, the United States Food and Drug Administration approved lifileucel, a Tumour-Infiltrating Lymphocytes (TILs) therapy for selected metastatic melanoma populations following a phase two single-arm trial which demonstrated objective response rates of 31.5%.^32^ Similarly, in a phase one/two trial of GD2-CART01 in neuroblastoma patients, CAR T-cell persistence was reported in up to 30 months after infusion with an overall response rate of 63%.^33^ These figures, although from different diseases and treatment modalities, can provide broad benchmarks in which future CAR T-cell studies of melanoma and other solid cancers may be assessed. For this systematic review, several limitations need to be acknowledged. A broad definition of CAR T-cell therapy was applied, creating heterogeneity within studies. Even so, this was deliberately conducted to capture as much evidence as possible across early CAR T-cell therapies. Therefore, in conducting this, the results of this review may not be applicable to traditional CAR T-cell platforms. Future reviews should focus on stricter definitions of CAR T-cell therapies, reducing heterogeneity and increasing clinical viability. With a pooled population of fifteen patients across five studies, including multiple single-patient and heterogeneous studies, there is a lack of large-scale clinical trials and a small number of studies reducing study quality. Due to this review’s small-scale studies, its results have limited direct clinical applicability. To overcome this issue, further clinical trials, ideally beyond phase one testing, need to be conducted which recruit more patients to achieve more robust results. At present, many such clinical trials are being conducted such as: NCT02830724, NCT06739226, NCT07193966, NCT04119024 with hopefully promising results. At present, adoptive cellular technologies for melanoma remain in its infancy. Future studies, including randomised controlled trials, comparing CAR T-cell therapy with other adoptive treatments, such as TILs, are required to better understand which approach has greater evidence or clinical effect. Similarly, long-term follow-up of survivors, with analysis of factors including long term adverse effects such as replication competent retrovirus, and clinical endpoints such as overall survival, are limited in this review. These would be key areas for future research to focus on.

The limitations of the included studies captured in this review need to also be acknowledged. Though, according to the updated draft ROBINS-I framework, most studies scored a low risk of bias, the study quality of this review is hindered by the inclusion of Fulbright et al (2020).^21^ This study published its results in a meeting abstract with minimal information available, and consequently, scored a serious risk of bias, severely damaging the quality of this review. With CAR T-cell therapy for the treatment of melanoma is still in its infancy, this review aims to capture all relevant evidence of published clinical trials to help inform future studies and reviews. Additionally, in accordance with the Cochrane Handbook,^15^ which advocates including as much relevant evidence as possible, including conference abstracts, as highly desirable, and to reduce risk of publication bias. For these reasons, Fulbright et al (2020)^21^ was ultimately included in this review. Of further note, this review may help guide researchers in future studies by building evidence towards GD2 as a viable cell target, supporting Gargett et al (2024),^23^ and its findings on persistence and expansion are also the highest recorded across all studies. However, due to this grading, ultimately conclusions based on this study from our review are especially uncertain and need to be interpreted with caution. Future reviews should focus on inclusion of new, more rigorous studies (once they have been completed) to reduce bias and for greater clinical applicability.

## Conclusion

CAR T-cell therapy for the treatment of melanoma represents a novel approach with preliminary clinical evidence that warrants further testing. This review synthesises current understandings amongst the available clinical data, highlighting trends and limitations for future studies and reviews to build upon.

## Supplemental Material

Supplemental Material - Chimeric Antigen Receptor T-cell Therapy for the Treatment of Melanoma: A Systematic Review of Phase One Clinical Trials Supplemental Material for Chimeric Antigen Receptor T-Cell Therapy for the Treatment of Melanoma: A Systematic Review of Phase One Clinical Trials by Pavan Shet, Prerana Ghosh, Karan Varshney and Sameen Sawdagar in Technology in Cancer Research & Treatment.

Supplemental Material - Chimeric Antigen Receptor T-cell Therapy for the Treatment of Melanoma: A Systematic Review of Phase One Clinical Trials Supplemental Material for Chimeric Antigen Receptor T-Cell Therapy for the Treatment of Melanoma: A Systematic Review of Phase One Clinical Trials by Pavan Shet, Prerana Ghosh, Karan Varshney and Sameen Sawdagar in Technology in Cancer Research & Treatment.

## Data Availability

All data is available upon request.*
