# Supplementary material for: Chimeric Antigen Receptor T-Cell Therapy for the Treatment of Melanoma: A Systematic Review of Phase One Clinical Trials
Source: Technol Cancer Res Treat. 2026 Jun 17;25:15330338261452884. doi: 10.1177/15330338261452884 (PMC13291571; doi:10.1177/15330338261452884)
Supplement: Supplemental Material - Chimeric Antigen Receptor T-cell Therapy for the Treatment of Melanoma: A Systematic Review of Phase One Clinical Trials [file sj-pdf-1-tct-10.1177_15330338261452884.pdf]

Supplementary Table 1: Search terms across databases

**Embase/medrxiv:**	(Chimeric Antigen Receptor OR CAR T OR “chimeric antigen receptor T-cell immunotherapy”) AND (melanoma OR "melanoma")
**Pubmed/Clinical trials/cochrane/International Clinical Trials Registry Platform (ICTRP):**	(Chimeric Antigen Receptors OR CAR T cells OR CAR T OR "CAR T cell therapy" OR "CAR T cell immunotherapy" OR "chimeric antigen receptor T cell therapy" OR "chimeric antigen receptor T cell immunotherapy" OR "chimeric antigen receptor" OR "engineered T cell" OR "modified T cell" OR "autologous T cell") AND (melanoma OR "skin cancer" OR "skin neoplasms" OR "malignant skin cancer" OR "malignant skin neoplasm")
